# Peer Review of “A Local Community-Based Social Network for Mental Health and Well-being (Quokka): Exploratory Feasibility Study”

**DOI:** 10.2196/33932

**Published:** 2021-10-27

**Keywords:** local social network, community health, well-being, digital health, consumer health

*This is a peer-review report submitted for the paper “A Local Community-Based Social Network for Mental Health and Well-being (Quokka): Exploratory Feasibility Study.”*

## Round 1 Review

### General Comments

I appreciate the opportunity to review this manuscript [1]. I hope that my feedback will help to strengthen the paper.

This paper has a strong theoretical background and empirical data. Overall, the manuscript is well prepared; however, it requires some major and minor corrections.

### Specific Comments

#### Major Comments

1. The value of this work seems to be significant.

2. The *Results* section should start with a data analysis. If Table 1 and “User Statistics” represent a sample, then the appropriate subheading should be provided.

3. In the *Discussion*, start your discussion with a short summary of what the main finding(s) of this study was/were. This section is too structured but at same time is confusing about the criteria of discussing the findings. I would suggest restructuring the way the data is discussed.

4. The subheadings in the *Methods* section should be related to the subheadings in the *Results* section.

#### Minor Comments

1. Minor technical/language corrections are needed (eg, “There is a strong, well-researched connection between social influence, social media, and health and wellness [2-3]”; “Try 3 different types of exercise this week”).

2. Double-check the style of the references, which should be prepared according to journal requirements (eg, references 22, 23, 28).
